# Variational Pair-Density Functional Theory: Dealing with Strong Correlation at the Protein Scale

**DOI:** 10.1021/acs.jctc.3c01240

**Published:** 2024-01-13

**Authors:** Mikael Scott, Gabriel L. S. Rodrigues, Xin Li, Mickael G. Delcey

**Affiliations:** † Division of Theoretical Chemistry and Biology, School of Engineering Sciences in Chemistry, Biotechnology and Health, KTH Royal Institute of Technology, SE-100 44 Stockholm, Sweden; ‡ Division of Theoretical Chemistry, Department of Chemistry, Lund University, SE-221 00 Lund, Sweden; § PDC Center for High Performance Computing, KTH Royal Institute of Technology, SE-100 44 Stockholm, Sweden

## Abstract

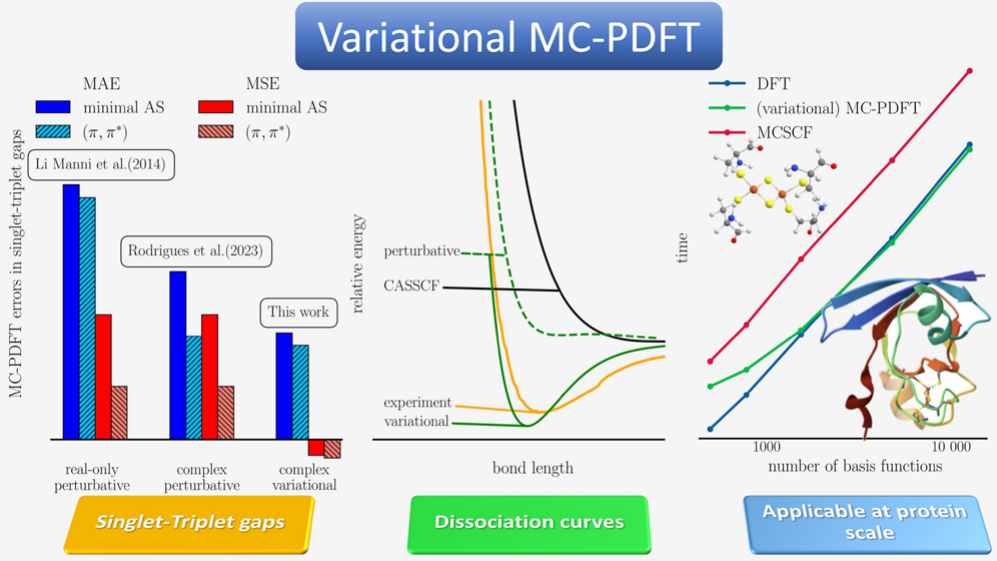

Multiconfigurational pair-density functional theory (MC-PDFT) offers a promising solution to the challenges faced by traditional density functional theory (DFT) in addressing molecular systems containing transition metals, open-shells, or strong correlations in general. By utilizing both the density and on-top pair-density, MC-PDFT can make use of a more flexible multiconfigurational wave function to capture the necessary static correlation, while the pair-density functional also includes the effect of dynamic correlation. So far, MC-PDFT has been used after a multiconfigurational self-consistent field (MCSCF) step, using the orbitals and configuration interaction coefficients from the converged MCSCF wave function to compute PDFT energies and properties. Here, instead, we propose to perform a direct optimization of the wave function using the pair-density functionals, resulting in a variational formulation of MC-PDFT. We derive the expressions for the wave function gradient and illustrate their similarity to standard MCSCF equations. Furthermore, we illustrate the accuracy on a set of singlet–triplet gaps as well as dissociation curves. Our findings highlight one of MC-PDFT’s standout features: a reduced dependency on the active space size compared to conventional multiconfigurational wave function methodologies. Additionally, we show that the computational cost of MC-PDFT is potentially lower than MCSCF and often on-par with standard Kohn–Sham DFT, which is demonstrated by performing a MC-PDFT calculation of the entire ferredoxin protein with 1447 atoms and nearly 12 000 basis functions.

## 1 Introduction

Density functional theory (DFT) is currently by far the most widely used method to compute the electronic structure of molecular systems, due in no small part to its high accuracy to cost ratio, scaling formally lower than Hartree–Fock (HF).^1,2^ While technically an exact formalism, in practice, DFT relies on approximations of the universal exchange–correlation functional. Over time, many approximations have been devised, addressing the difficulties that previous functionals had with specific classes of problems, for example, long-range van der Waals (or dispersion) interactions^3,4^ and charge-transfer states.^5,6^ However, within the Kohn–Sham (KS) formalism, no satisfying answer has been found to the “strong correlation problem”, typically present in systems such as transition metals, open-shells, and in bond-breaking scenarios.^7^

Instead, most approaches meant to address this issue rely either fully on wave function methods, such as the multiconfigurational self-consistent field (MCSCF),^8,9^ or beyond Kohn–Sham using some form of hybrid method that combines DFT with a multideterminantal wave function.

Early ideas, already in the 1970s, suggested simply adding a correlation functional to a MCSCF energy.^10^ The idea was that the DFT correlation functional would only treat the dynamical correlation, while MCSCF would treat only the strong correlation. However, the latter assumption is poor as MCSCF also includes dynamical correlation within the active space, leading to the double counting problem.^11^

Since then, various approaches have been suggested to avoid double counting. Some approaches attempt to estimate the DFT correlation already contained within the active space and remove it from the DFT part.^12−15^ Another very popular approach is to use range-separation, grounded in the idea that dynamical correlation is mostly short-ranged, and well reproduced by DFT, while MCSCF should properly describe electronic interactions at long-range.^16−18^

A fundamentally different approach has been to completely forego the MCSCF energy opting instead to use a functional of the density and on-top pair-density in-place of the usual spin-density functionals.^19,20^ The resulting multiconfigurational pair-density functional theory (MC-PDFT) is formally free from double counting as the energy comes solely from the pair-density functional (except for the kinetic energy as in Kohn–Sham). Typically, the pair-density functionals are not developed from the ground up, instead standard spin-density functionals are translated to employ the on-top pair-density.^19,21−23^ These translated functionals, while congruent with the energies of KS-DFT for systems described by a single determinant, demonstrate superior accuracy for systems characterized by multiple determinants.^20−22^

The most popular implementation of MC-PDFT is that of Li Manni et al.^19^ from 2014. In this implementation, one first performs a MCSCF calculation, and once converged, the density and pair-density from the MCSCF wave function are used to compute the MC-PDFT energy in a post-SCF manner. This means the orbitals and configuration interaction (CI) coefficients were not optimized under the influence of the PDFT functional. This can be thought of as a first-order perturbation going from MCSCF to a fully optimized MC-PDFT wave function, and as such, in the rest of the paper, we will denote this approach “perturbative”.

There are several reasons one may want to instead derive a formulation where the wave function is variationally optimized to minimize the PDFT energy. First, such an optimization would ensure that the densities are optimized under the influence of the dynamical correlation, which we can expect to be more accurate (we note, however, that the use of HF orbitals to compute DFT energies has been suggested as a superior choice for some properties^24^). This is also a more well-defined scheme to formulate a Hohenberg–Kohn equivalent for MC-PDFT since such a theorem assumes we have the exact densities that the MCSCF ones clearly are not. Furthermore, a variational energy provides significant simplifications to compute properties, in particular, molecular gradient or spectroscopy using response theory. For example, the MC-PDFT analytical gradient implementation of Sand et al. requires the costly evaluation of the wave function response even for single-state optimization,^25^ while these terms disappear if the wave function is variationally optimized.

In this article, we derive the wave function gradients for a variational MC-PDFT implementation. We illustrate how such gradients can be cast in a form that is readily useable within standard MCSCF codes, albeit with some pitfalls. We discuss the computational cost of the various steps of this implementation and show that it has the potential of being significantly more efficient than standard MCSCF. We then examine the accuracy of variational MC-PDFT on some dissociation curves and singlet–triplet gaps using the translated local density approximation (tLDA) level and translated generalized gradient approximation (tGGA) functionals. We continue with a discussion on active space convergence and how it relates to the question of double counting. Finally, we illustrate of the efficiency of the method, and our implementation into the MultiPsi package,^26,27^ by performing the calculation of an entire protein at the MC-PDFT level, far exceeding any precedent record for largest multiconfigurational calculation ever performed.

## 2 Theory

### 2.1 Motivation for Pair-Density Functional Theory

The use of the density and on-top pair-densities instead of the usual spin densities is motivated by the deficiency of the latter in correctly describing fundamental properties, such as bond dissociation or the degeneracy of the three triplet components. This issue is specifically related to the use of spin densities since using only the total density as argument does not lead to such a problem. For example, the three components of the triplet have the same total densities and thus the same energy in DFT, but different spin densities and thus potentially different energies in the more commonly used spin-DFT.

However, functionals designed using only the total density are not as accurate for open-shell systems and, for example, would struggle to distinguish an open-shell singlet and the corresponding triplet.

Using the total density and on-top pair-density solves both problems. While the three triplet components have the same densities and pair-densities, the corresponding open-shell singlet has a similar density but a very different on-top pair-density. Similarly, bond-dissociated fragments have the same density and pair-density as each fragment separately. This means that by construction, PDFT solves the strong correlation issue. Some authors have even argued that spin-DFT actually works because it approximates PDFT in single determinant cases.^28^

### 2.2 Pair-Density Functional Translation

While in principle it would be desirable to derive directly a pair-density functional (PDF) expression from first-principles, currently, most pair-density functionals are derived by “translating” existing spin density functionals (SDFs).

The idea is that for the case of a single determinant the total density ρ and the on-top pair-density Π share a simple relation with the usual spin densities ρ_α_ and ρ_β_
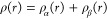
1and
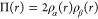
2

With these relations, it is possible to translate existing SDFs to use the total and on-top pair-densities while still ensuring they yield the same results for the single determinant case. The simplest way to achieve this is to invert eqs 1 and 2 to compute the “effective” spin densities from ρ and Π
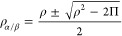
3

In a previous article, we illustrated how doing this sometimes leads to “effective” spin densities that are complex.^23^ However, this still leads to a final energy contribution that is real, and we argued that the resulting energy has the expected physical behavior. In this article, we use these translated variants of LDA, PBE, and BLYP, including the complex regime, which we denote ctLDA, ctPBE, and ctBLYP.

### 2.3 Energy Expression

For MC-PDFT, the energy can be written as
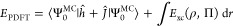
4We note that this is simply a generalization of Kohn–Sham theory using a multiconfigurational wave function and a pair-density functional.

The density and on-top pair-densities can be computed from the one- (1-DM) and two-body density matrices (2-DM), respectively. The total density poses no problem as it can be computed from the standard density matrix *D*_μν_ in atomic orbitals (AO) basis, as is usually done in DFT. On the other hand, while the active 2-DM is always computed in a MCSCF calculation, the on-top pair-density requires in principle the total 2-DM and the storage of this quantity, whether in AO or MO basis, is dissuasive for any medium to large molecular system. However, the inactive parts can be computed directly from the inactive density since it satisfies the equation eq 2 for a single determinant. Doing this would still require separately computing the inactive density, which is a non-negligible added cost.

For efficiency, we then suggest a slightly variable change. Instead of using the on-top pair-density, we propose using a new quantity which we call the “correlated” on-top pair-density, Π̃(*r*), and which we define as
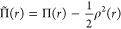
5

With this definition, it is easy to see that for a single closed-shell determinant, Π̃(*r*) = 0, and hence one can argue that this variable indeed measures correlation.

The advantage of using this variable is that it can be computed directly from the “non-separable” Γ_*pqrs*_^ns^ part of the 2-DM Γ_*pqrs*_
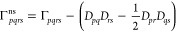
6This formula simplifies for all but active orbitals, and it thus does not depend at all on the inactive orbitals. Additionally, this intermediate is often already present in MCSCF codes, as it can be useful for the computation of geometrical gradients.

With the new definition in eq 7, the translation formula of eq 3 simplifies to
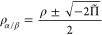
7

Now, to compute Π̃(*r*) on the grid, we only need to compute the active MOs on the grid, which are in most cases inexpensive due to their small number, and multiply those by the nonseparable active 2-DM in MO basis, which is also in general small enough to be stored in memory without any issue. The total density is computed as usual on the AO basis.

To simplify notations, we are dropping the tilde in the rest of the article and simply denote Π the correlated pair-density.

### 2.4 Wave Function Gradient

To derive the gradient of eq 4, concerning any wave function parameter *X*, we begin with the *E*_xc_(ρ, Π) term
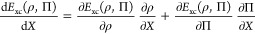
8where, in the molecular orbital (MO) basis, the densities ρ and Π are defined as
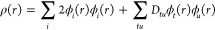
9and
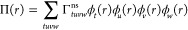
10We have used here the conventional notation of *i*, *j* for inactive (occupied) MOs, *t*, *u*, *v*, and *w* for active MOs and *p*, *q*, *r*, and *s* for general indices.

We will define *v*_xc_ as the first term in eq 8, by analogy to the standard Kohn–Sham potential, while we will denote *w*_xc_ the second term, which is the only two-electron component of MC-PDFT.

The gradient then essentially resembles that of a modified Hamiltonian, with integrals defined as
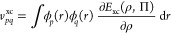
11and
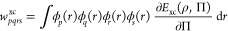
12

With the above equations established, we can delve into the explicit terms for both the orbital and CI gradients. The PDFT energy depends on both orbital and CI parameters, represented by κ_*pq*_ and *C*_*i*_. Due to the use of the MO basis, the dependence on the orbital coefficients is embedded within the integrals. As a result, the orbital gradient can be expressed as a Fock matrix, with different expressions depending on whether the first index is inactive or active
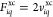
13and
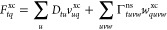
14These formulations bear a resemblance to conventional MCSCF gradients. In this context, *v*^xc^ contributes to the inactive Fock matrix, while the term with *w*^xc^ plays a role analogous to a *Q*-matrix. For computational efficiency, MultiPsi calculates the *v*^xc^ term in the atomic orbital (AO) basis, while the *w*^xc^ term maintains three active (MO) indices accompanied by one AO index.

Let us now move to the derivatives, with respect to the CI coefficients. Those come in the form of effective integrals to provide the CI code. The two-electron integrals are simply the *w*^xc^. However, the one-electron integrals contain contributions from both *v*^xc^ and *w*^xc^ due to the 1-body density matrix terms in the nonseparable 2-particle density matrix. Therefore
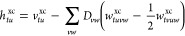
15Note that due to the symmetry of *w*^xc^ with permutations of the indices, .

### 2.5 Classical Terms

The above equations contain pure contributions from the exchange–correlation term. We now add classical contributions. First, for the orbital gradient, we define an inactive Fock matrix
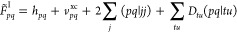
16such that
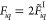
17and
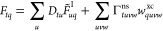
18

Note that we can reorganize terms to increase the similarity to standard MCSCF wave function gradient even further by defining an active Fock matrix
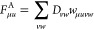
19and then defining a new inactive Fock matrix as
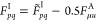
20

The effective Fock matrix for the orbital gradient thus becomes
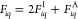
21and
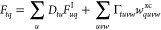
22Note the use of the full active two-particle density matrix in this last expression. This allows us to implement variational MC-PDFT optimization in a straightforward way in an existing MCSCF code.

With this definition, the CI code requires *F*_*tu*_^I^ as one-electron integrals and *w*_*tuvw*_^xc^ as two-electron integrals, as there are no classical explicit two-electron contributions.

### 2.6 Implementation

The equations above have been implemented in the MultiPsi^26^ MCSCF code as well as the core DFT routines in VeloxChem.^29^ The MCSCF module uses a two-step quasi-Newton orbital optimization that required no changes beyond replacing the MCSCF integrals by the PDFT equivalents. The approximate Hessian is already defined in terms of the Fock matrices and thus requires no change. The resulting approximate Hessian does not include any terms depending on the second derivative of the PDFT functional, but it is typically acceptable and remedied by the Hessian update. Overall, during our test calculations, we found that the number of iterations needed to converge was essentially unchanged between the MCSCF and MC-PDFT.

However, the configuration interaction step requires changes. In MultiPsi, the CI is solved by using a standard Davidson approach, but this approach usually fails when combined with PDFT integrals. The issue is that the integrals discussed here provide only a first-order approximation of the energy around the starting configuration. We instead wrote a quasi-Newton CI optimization that requires re-evaluating the integrals at each CI iteration. This is most likely not optimal, and we will address this in future work.

Note that any MCSCF code using a super-CI algorithm for orbital optimization would likely suffer from similar issues even in this step.

### 2.7 Computational Cost

In a two-step MCSCF optimization, as in MultiPsi, the computational cost of MCSCF is essentially the sum of the cost of the integral calculation and the CI step.

The CI costs are virtually identical between MC-PDFT and MCSCF. The most expensive step in both cases is the computation of the **σ** = **HC** vector. This scales factorially with the active space size, meaning that for a reasonably sized molecule and small active spaces [typically below CAS(14,14)] the CI part tends to be a negligible part of the total cost, while it dominates for larger CI expansion.

For the integrals, MC-PDFT is dominated by the Coulomb matrix calculation and DFT integration. Conventional integral calculations lead to a formal *N*^4^ scaling for the former with *N* the size of the atomic orbital basis, but the actual scaling is typically much lower due to screening, and resolution of identity can also be used to reduce the scaling further. In the DFT integration, the most expensive step is the calculation of the gradient terms described in eqs 11 and 12. The former has a formal scaling of *N*^2^*N*_grid_ with *N*_grid_ the size of the DFT integration grid, as in standard Kohn–Sham DFT. In the latter, since we only need the terms with three active orbitals *w*_*puvw*_^xc^, the scaling becomes *NA*^3^*N*_grid_ with *A* the number of active orbitals which is typically small. Assuming *A* ≈ 10, this term will cost about the same as the standard Kohn–Sham *v*_xc_ for a number of basis function *N* ≈ 1000 which is a medium size calculation. Note that the calculation of the active molecular orbitals and the pair-density on the grid scale, respectively, are *NAN*_grid_ and *A*^4^*N*_grid_ which are thus indeed inexpensive compared to the aforementioned costs. Moreover, DFT integration can in practice be done in even-sized grid batches, such that the overall scaling become asymptotically linear with respect to increasing number of batches, where the number of significant basis functions for each grid batch remains almost constant on average due to efficient screening.^30^

This computational cost can be compared to a standard MCSCF for which the most expensive step (outside of CI) requires the transformation of the AO integrals to the active MO basis for a dominant scaling of *AN*^4^. In other words, variational MC-PDFT is cheaper than MCSCF. Actually, for any reasonable size system with small to medium active spaces, the cost of variational MC-PDFT is about the same as that of a standard Kohn–Sham DFT. The different formal scalings are summarized in Table 1. The possibility to treat strong correlation within DFT with essentially no extra cost is, in our opinion, one of the most appealing qualities of the method.

**Table 1:** Formal Dominant Scaling of Different Steps of KS-DFT, MCSCF, and Our Variational MC-PDFT

method	DFT integration	integrals	CI
(pure) KS-DFT	*N*_grid_*N*^2^	*N*^3^	
(pure) MC-PDFT	*N*_grid_(*N*^2^ + *A*^3^*N*)	*N*^3^	factorial
MCSCF		*AN*^4^	factorial

However, we note that this discussion is restricted to pure functionals since, if a fraction of MCSCF energy is added, as has been suggested by some authors,^31,32^ the cost of both variational and perturbative approaches are naturally going to be similar to that of MCSCF.

## 3 Computational Details

The singlet–triplet gaps were computed using the cc-pVTZ basis set^33,34^ and the dissociation curves were computed using the def2-TZVP^35^ basis set at the MC-PDFT levels of theory. All MC-PDFT and CASSCF calculations were carried out in the MultiPsi package.^26^ The CASPT2^36^ calculations were performed using OpenMolcas.^37^ The standard IPEA shift of 0.25 Ha was used (including for Cr_2_, explaining the worst results than those presented in ref (38)). An imaginary shift of 0.1 Ha was used for Mg_2_, and 0.3 Ha for the metal dimers due to the presence of strong intruder states. For these CASPT2 calculations, the Dunning cc-pVQZ basis set was employed.

## 4 Results

In this section, we look at the qualitative and quantitative behavior of variational MC-PDFT compared to the perturbative approach. This is not meant to be an extensive benchmark, and a more thorough analysis for a wider range of systems and functionals will be presented in a future publication. We also do not include results computed using the “ft” functionals.^38^ While these functionals include the gradient of the pair-density (which our “ct” functionals neglect), they also significantly modify the behavior around Π = 0 (that is the closed-shell case), and it thus becomes difficult to properly interpret their successes or failures.

### 4.1 Dissociation Curves of Diatomic Species

One of the most common ways to benchmark multiconfigurational methods is by analyzing dissociation curves. Here, we look at a few examples of dissociation curves of systems that, while requiring multireference treatment, also include a significant amount of dynamical correlation. More specifically, we chose Cu_2_, Cr_2_, and Mg_2_ which have been the subject of previous studies due to their multireference character.^38,40−42^ We computed each dissociation curve at the MC-ctPBE and MC-ctBLYP levels of theory using both the perturbative and variational approaches as well as the CASSCF and CASPT2 levels of theory.

**Figure 1:**
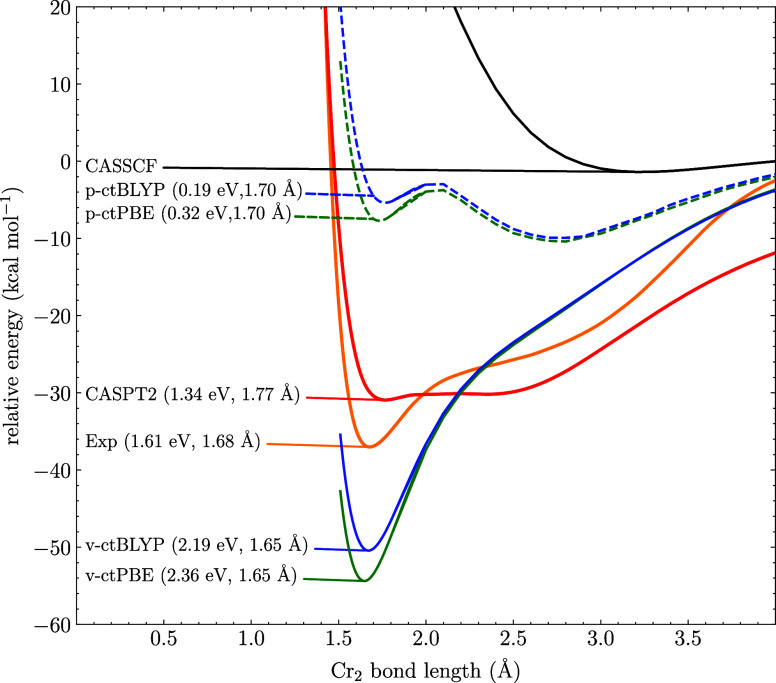
CASSCF, CASPT2, MC-ctPBE, and MC-ctBLYP dissociation curves of Cr_2_ computed with a (12,12) active space and the def2-TZVP basis set. The p- and v-prefixes denote perturbative and variational approaches. The dissociation energies and equilibrium bond lengths are written in the parentheses. The experimental curve is adapted from ref (39).

We start with the dissociation of Cr_2_ which famously exhibits a very strong multireference character with the HF-wave function accounting for ∼50% at equilibrium.^40^ An appropriate active space for this molecule includes both 3d and 4s orbitals, resulting in a CAS(12,12). As can been seen in Figure 1, the experimental dissociation energy is 1.61 eV and at a higher distance there is a distinctive shoulder feature. Here, CASSCF completely failed to predict any binding. The CASPT2 energy is, on the other hand, close to the experiment, though the results are very dependent on both the IPEA and imaginary shifts. For MC-ctPBE and MC-ctBLYP in their perturbative forms, a heavily underestimated binding energy of 0.19 and 0.32 eV, respectively, are predicted though the shoulder feature at higher separation is predicted to be lower in energy than the first minimum. For the variational approach, there is a clear improvement in the dissociation energies compared to experiment, though with significant overbinding, here calculated as 2.36 and 2.19 eV for MC-ctPBE and MC-ctBLYP, respectively. The equilibrium bond distance is also reasonably well predicted by variational MC-PDFT.

**Figure 2:**
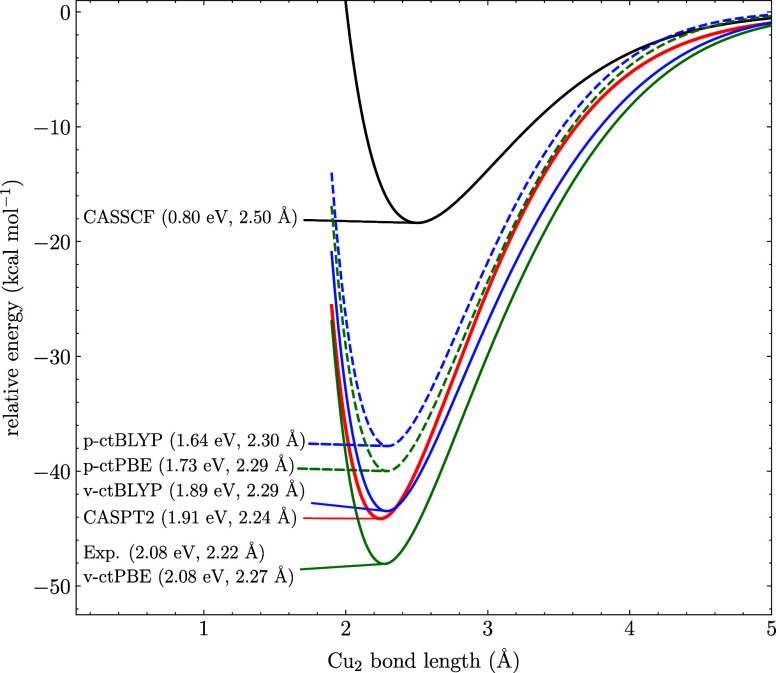
CASSCF, CASPT2, MC-ctPBE, and MC-ctBLYP dissociation curves of Cu_2_ computed with a (2,2) active space and the def2-TZVP basis set. The p- and v-prefixes denote perturbative and variational approaches. The dissociation energies and equilibrium bond lengths are written in the parentheses. The experimental reference is taken from refs (43) and (44).

For the copper dimer Cu_2_, the experimental dissociation energy is measured to 2.08 eV.^44^ All methods correctly predict the presence of a bond, even when employing a minimal (2,2) active space in this case, as shown in Figure 2. Specifically, CASSCF predicts a bond energy of 0.80 eV, which is improved by the perturbative MC-ctPBE and MC-ctBLYP at 1.64 and 1.73 eV, respectively. Next, the variational methods improves the results further at 1.89 and 2.08 eV for MC-ctBLYP and MC-ctPBE, respectively. This indicates the importance of orbital relaxation near equilibrium distance. When comparing the equilibrium bond lengths, the variational methods are consistently closer to the experimental measurement as compared to their perturbative counterparts, although the error at the CASPT2 level is lowest. However, all variational methods are at or near CASPT2 quality in predicting the dissociation energy.

**Figure 3:**
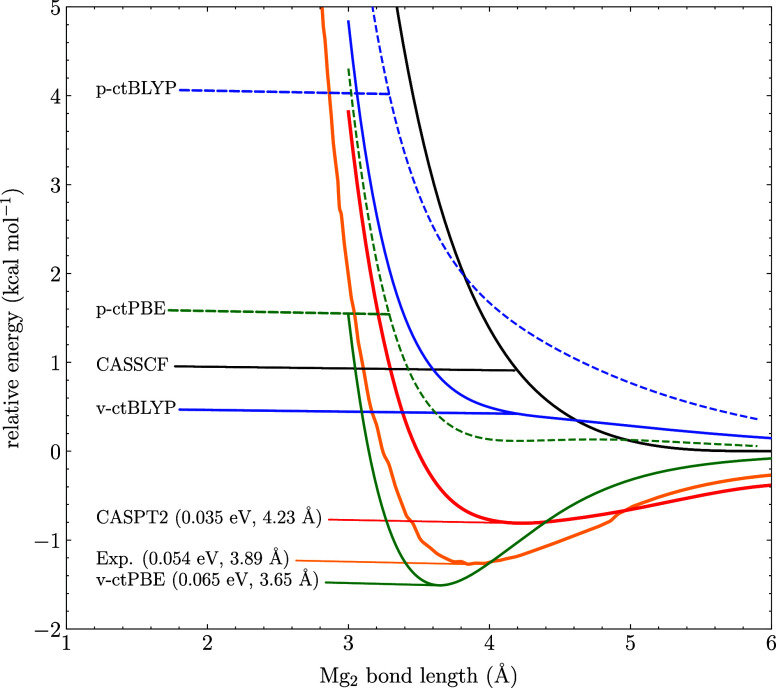
CASSCF, CASPT2, MC-ctPBE, and MC-ctBLYP dissociation curves of Mg_2_ computed with a (4,8) active space and the def2-TZVP basis set. The p- and v-prefixes denote perturbative and variational approaches. The dissociation energies and equilibrium bond lengths are written in the parentheses. The experimental reference is taken from ref (45).

We also looked at the magnesium dimer, which is a very different test case as it dissociates into closed-shell fragment and present only a weak bond, experimentally determined at 0.054 eV.^45^ There is, however, some strong correlation between the occupied 3s and the 3p orbitals. An adequate active space for this molecule should be including the 3s^2^ electrons and the 3p orbitals resulting in (4,8) active space. At the CASSCF level of theory, no bond is predicted to exist, as shown in Figure 3. The same is true for MC-ctBLYP in both the variational and perturbative forms, though the variational potential is less repulsive. At the CASPT2 level, a dissociation energy of 0.035 eV is predicted which is in reasonable good agreement with the experiment. Lastly, at the MC-ctPBE level of theory, the best fit with the experiment is found at 0.065 eV, i.e., within 0.2 kcal·mol^–1^ with respect to the experiment. Again, in this case, the variational approach outperforms the perturbative. For such weak bonds, high errors in bond lengths are to be expected, but the predicted bond length at the variational MC-ctPBE level is slightly better than the CASPT2 one, although both have an error higher than 0.2 Å.

### 4.2 Singlet–Triplet Gaps

The singlet–triplet energy gaps for a diverse set of molecules were computed using the ctLDA, ctPBE, and ctBLYP functionals as well as their real-only translated forms, with either a minimal (2,2) active space or including the entire π system for the molecules where it is relevant. For the complex-translated functionals both the variational and perturbative methodologies were computed. The molecules in this singlet–triplet-benchmark are adapted from our previous work and consist of 13 molecules including both molecules that adhere to Hund’s rule or violate it.^23^ For a more accurate representation of these singlet–triplet gaps, the cc-pVTZ basis set is used here, in contrast to the cc-pVDZ basis set used in our previous work. In addition, in the previous work, for some molecules, such as O_2_, a single-state MCSCF optimization (and thus the perturbative MC-PDFT) for small active spaces sometimes found an incorrect singlet state as the lowest state, which we then fixed by using state-averaged MCSCF over the two degenerate singlet states. Here, we did not do that, and thus, some results differ significantly from the previous work. We note that this is one of the advantages of using a variational MC-PDFT approach, as correlation is included in the wave function optimization, and thus, the correct ground state is more likely to be found automatically.

**Figure 4:**
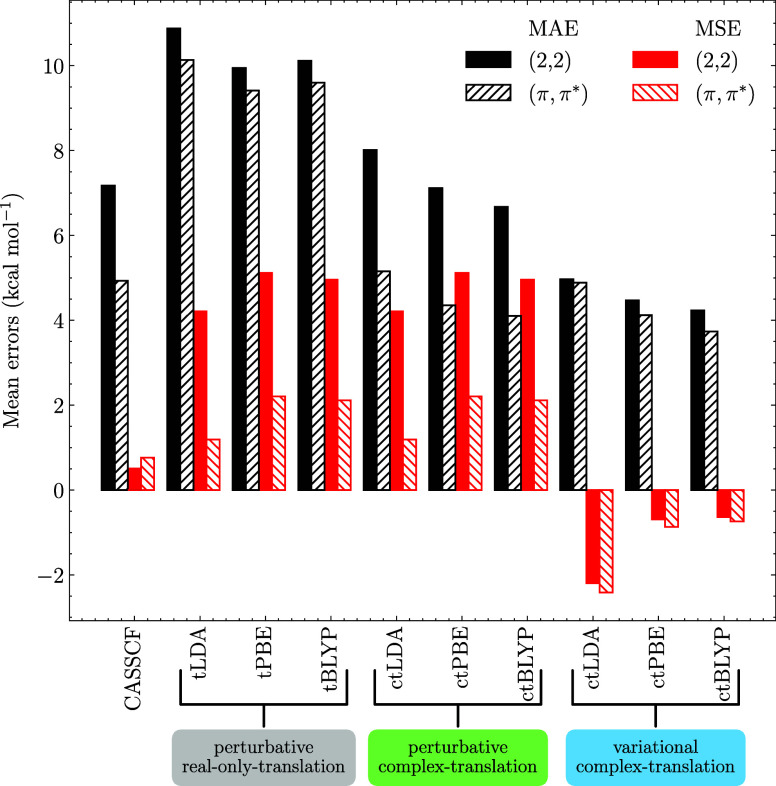
MAE and MSE total errors (kcal·mol^–1^) of singlet–triplet energy gaps of Hund and non-Hund rule molecules (ref (23)) calculated with the perturbative MC-tLDA, MC-tPBE, and MC-tBLYP levels of theory (similar methodology as in ref (46)) and MC-ctLDA, MC-ctPBE, and MC-ctBLYP levels with the perturbative and variational approach as well as at the CASSCF level. The cc-pVTZ basis set was used. The reference values are either doubly electron-attached coupled-cluster reference (ref (47)) or experimental reference for O_2_ (ref (48)).

The results exhibit a steady improvement going from the real-only perturbative results to the complex translation and finally the variational results; see Figure 4 as well as the Supporting Information for the individual numerical values. The real-only translated functionals produce ST gaps with a mean absolute error (MAE) of more than 10 kcal·mol^–1^ for all functionals. Employing the complex-translation already lowers the MAE to around 8 kcal·mol^–1^ for the minimal active spaces and 4 kcal·mol^–1^ for the larger spaces. By contrast, for the variational methods, both the minimal and large active spaces display MAE around ∼4 kcal·mol^–1^. The mean signed error (MSE), i.e., accounting for under- and overestimation of the singlet–triplet gaps, further indicates that the perturbative approaches consistently overestimate the singlet–triplet gap, whereas the variational approach is close to zero, neither under- nor overestimating the energies. Looking in details in the Supporting Information, we see that this is because of opposite trends for Hund and anti-Hund molecules. Unfortunately, neither perturbative nor variational MC-PDFT successfully predicts the reverse singlet–triplet gap of most anti-Hund molecules, which is likely an issue with the functionals themselves.

Overall, the variational approach is the only one in this small test set that consistently improves upon the CASSCF, especially with the tGGA functionals. Expanding the size of the active space shows only modest improvements in the MAE for the variational approach, which is in stark contrast with the perturbative results,^48^ showing that the method has a reduced active space dependency.

### 4.3 Electronic Structure Properties: Dipole Moments

One of the advantages of our variational implementation is that it allows a relaxation of the wave function under the influence of the dynamical correlation, potentially leading to a more accurate density. One way to quantify that is by looking at the predicted dipole moment. Hait and Head-Gordon performed an extensive benchmark of dipole moments for small molecules comparing DFT to accurate coupled cluster results.^49^ They also showed a smaller set of 11 molecules that were particularly sensitive to the method. We calculated 7 of these molecules with MCSCF, BLYP, and our variational MC-ctBLYP. All calculations were done with the aug-cc-pVTZ basis set. Note that for the “perturbative” MC-PDFT, the dipole would be the one predicted by the MCSCF wave function. The four removed molecules were fully single-determinantal cases for which no reasonable active space could be chosen, and thus, our results would simply match the corresponding Hartree–Fock and BLYP results.

The errors of our calculations compared to those of the reference are shown in Table 2. In almost all cases, DFT significantly improves over MCSCF. This is not surprising as the active spaces are small compared to the total size, and it was found in the original article that DFT even with pure GGA functionals gets better dipole moments than Hartree–Fock. Interestingly, our MC-PDFT dipole moments are also almost consistently better than the DFT ones, with a mean absolute error going from 0.22 to 0.186 D. We can add that in most cases, the errors of MC-tBLYP and MCSCF were of opposite signs, showing that a hybrid functional may improve even further the results. Overall, this confirms that our variational optimization improves the wave function compared to MCSCF and thus the perturbative MC-PDFT.

**Table 2:** Error in the Dipole Moments (in debye) for MCSCF, BLYP, and MC-ctBLYP Compared to the Reference in Ref (49)

molecule	BLYP	MC-ctBLYP	MCSCF	active space
BF	0.19	0.10	0.27	(2,3)
HCCF	0.24	0.21	0.28	(4,4)
HCCCl	0.25	0.21	0.27	(4,4)
CF	0.22	0.20	0.38	(3,3)
BS	0.08	0.13	0.10	(5,5)
CF_2_	0.17	0.11	0.40	(2,2)
NOCl	0.38	0.34	0.69	(4,4)
MAE	0.22	0.19	0.34	

### 4.4 Double Counting and Active Space Dependency

As mentioned in the theory section, MC-PDFT has formally no double counting since we do not sum the MCSCF energy with a DFT contribution but instead use a Kohn–Sham-like expression. However, this statement may still be refined a little when using translated functionals, as we are in this work. Indeed, these functionals were typically designed for Kohn–Sham DFT and, as such, are meant to give the correct energy for a single determinant. Since we are using a variational framework with increased flexibility in our wave function, we are guaranteed to obtain a lower energy than the corresponding Kohn–Sham calculation with a single determinant, even if the functional was a translation of the exact functional, which could be considered double counting.

This caveat does not contradict our initial statement: MC-PDFT is formally free from double counting, and the potential errors described here simply indicate issues within the functionals and are not inherent to the method itself. If instead we were to design a pair-density functional from first principles, with the intent that with the exact densities and pair-densities we would obtain the exact energy, we would indeed avoid all double counting issues. However, the problem then becomes that we would, in principle, need a full CI expansion to obtain the correct energy, which defeats the purpose of using PDFT.

These dilemmas all link to the important concept of active space dependency, namely, how quickly does the energy converge with increasing active spaces toward the full CI limit. With a translated “exact” Kohn–Sham functional, any changes for increasing active spaces would stray us away from the correct energy, while with the “exact” PDFT functional, smaller active spaces would decrease the accuracy. Thus, in both cases, one would want a very small change in energy as we change the active space such that we could only use the minimal active space needed to properly describe the system and get an accurate energy.

**Figure 5:**
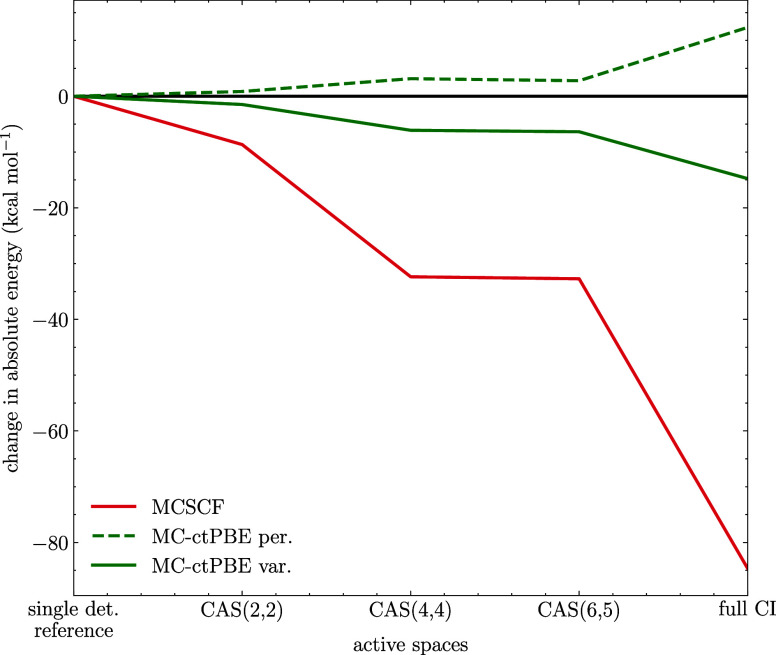
Changes in the absolute energies (kcal·mol^–1^) of a water molecule with increasing active space sizes for MCSCF and MC-ctPBE using the 6-31G basis set.

In Figure 5 we show the evolution of the absolute energies of water at equilibrium distance with the 6-31G basis with increasing active space size.

While, as expected, the variational MC-PDFT and MCSCF follow the same trends of decreasing energy with increasing active space size, it is clear that the sensitivity is much greater for MCSCF than MC-PDFT, with a change in energy from a single determinant to a full CI expansion of 85 kcal·mol^–1^ for MCSCF compared to 15 kcal·mol^–1^ for MC-ctPBE. Note that for the perturbative MC-PDFT, there is no requirement that the energy goes down with increasing active space sizes, and in this case, it actually consistently goes up instead. Still, in this example, both variational and perturbative formulations show a similarly reduced active space dependency compared to MCSCF. This contrasts with the results from Figure 4 which indicate that the variational formulation may be overall less active space dependent, at least when it comes to relative energies. The same phenomenon can also be seen with the occupation numbers. Looking for example at benzene with a CAS(6,6)/cc-pVDZ calculation, as shown in Table 3, it is clear that the MC-PDFT occupation numbers are closer to 2 and 0 which means that the CAS(6,6) result with MC-PDFT is closer to the single determinant result than for MCSCF. This also explains why the perturbative energy goes up as we increase the active space since the optimal densities are closer to that of a single determinant wave function.

**Table 3:** Natural Orbital Occupation Numbers at MCSCF and MC-ctPBE Levels of Theory of Benzene with a CAS(6,6) Active Space by Using the cc-pVDZ Basis Set

	MO-1	MO-2	MO-3	MO-4	MO-5	MO-6
CASSCF	1.961	1.902	1.902	0.100	0.100	0.036
ctPBE	1.995	1.992	1.992	0.008	0.008	0.005

This is reduced active space dependency is a very important property, not just because it makes the double counting dilemma less significant but also because in practice, it means a MC-PDFT calculation can probably reach good accuracy with a smaller active space than typically used in MCSCF and CASPT2/NEVPT2, which is in line with a previous work on MC-PDFT.^50^ In particular, we expect that problems like the need to include the double-shell effect in some metal complexes^51^ are not as relevant with MC-PDFT, increasing further the efficiency of this method compared to CASPT2/NEVPT2.

### 4.5 MC-PDFT at the Protein-Scale: Fe_2_S_2_ Ferredoxin

**Figure 6:**
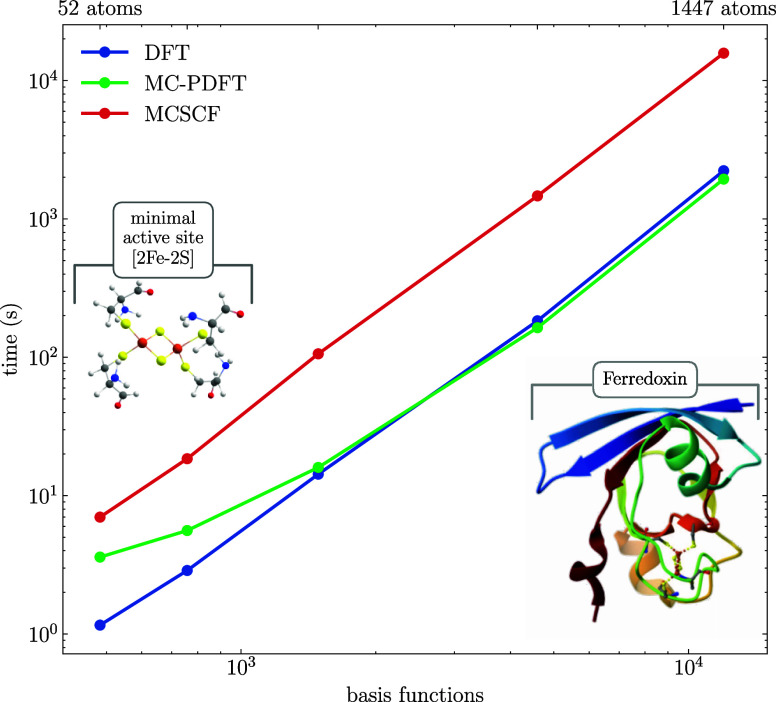
Computational time for a single integral evaluation of small to full models of the ferredoxin protein at the DFT, MC-PDFT, and MCSCF levels of theory using a CAS(10,10) active space for the multiconfigurational methods and the BLYP/ctBLYP functional for the DFT methods.

To illustrate the performance of the variational MC-PDFT, we performed an MC-ctBLYP calculation on several models of the oxidized form of ferredoxin and compared the computational performance to both standard MCSCF and unrestricted DFT with the BLYP functional. This protein contains a [2Fe–2S] complex with two antiferromagnetically coupled irons. The geometry is taken directly from the 1.3 Å resolution X-ray structure (pdb: 1QT9).^52^ The models were generated by progressively adding amino acids located within a specific distance from the [2Fe–2S] complex, giving us five models from 52 atoms to the entire protein and its 1447 atoms. Using the def2-SV(P) basis set, the number of basis functions ranges from 484 for our smallest model to 11 986 for the full protein. We chose a minimal active space of CAS(10,10), corresponding to the five singly occupied d-orbitals of each iron atom, coupling to a singlet state, which following our active space discussion in the previous section we can hope to be enough to accurately describe this system. The calculations were run with our programs VeloxChem and MultiPsi on 8 nodes consisting each of two 16-core Intel Xeon Gold 6130 CPUs, for a total of 256 cores. The resulting timings for one integral calculation (sum of the conventional integrals and DFT integration) are shown in Figure 6. We note that this does not include either the time for the CI iterations or the optimizer (DIIS for Kohn–Sham DFT and Quasi-Newton for MCSCF and MC-PDFT). For such a small active space, the former is only a couple of seconds; however, the latter can be non-negligible for the larger system sizes.

As can be seen in the figure, for the smallest models, the higher cost of the DFT integration in MC-PDFT can dominate and make the calculation more expensive than UDFT, though still cheaper than MCSCF. Running the smallest 52 atom model on a single node, KS-DFT takes 8.4 s per integral calculation, while MC-PDFT takes 12 s and MCSCF 45 s. In our implementation, the DFT integration dominates both DFT and MC-PDFT and most of the difference comes from the calculation of the *w*^xc^ term which takes about 2.3 s. However, since these terms depend mostly on the active space size, they become insignificant for larger systems, and for the two largest models, our MC-PDFT is actually slightly faster than DFT. The reason for this is that MC-PDFT only computes a single Coulomb Fock matrix, while unrestricted DFT requires two. Thanks to the efficient implementation, the cost is less than twice that of a single Fock matrix but still higher than for a restricted framework. For the largest system, the integral calculations for MC-PDFT is about 32 min, out of which the DFT numerical integration represents only 5%. That time is 37 min for KS-DFT and 4 h 34 min for MCSCF. The average scaling of all these calculations with the number of basis functions was about *N*^2.3^. The difference lies mostly in the prefactor, which is higher for MCSCF than for the DFT methods by a bit less than a factor of 10, approximately matching the number of active orbitals.

It is worth noting that these are, to our knowledge, by far the largest multiconfigurational calculations ever performed in terms of molecular size and number of basis functions, and the first time an entire protein was computed with such methods.

## 5 Conclusions

In this work, we introduced a fully variational formulation of MC-PDFT, which not only provides an improved framework for property calculations but also displays promising results in numerical applications, typically outperforming the “perturbative approach” that had been used so-far. In many cases, the results approach chemical accuracy, which is remarkable, as the functionals used are only pure functionals, which we know to be typically lacking for most applications.

Among the most important characteristics shown by this variational approach is a reduced active space dependency compared to that of normal MCSCF as well as perturbative MC-PDFT formulations. Furthermore, these results were obtained at a computational cost that is actually on-par with standard Kohn–Sham DFT for small to medium active spaces. In this sense, variational MC-PDFT really represents an extension of Kohn–Sham DFT to multiconfigurational wave functions. In addition, due to the reduced active space dependency, we expect that only the absolutely essential orbitals will be required in the active space, which are typically easier to predict and therefore may render the method more user-friendly and even arguably easier to use than broken-symmetry DFT.

The method now needs to be extended to compute molecular properties beyond the ground state energy, and we believe that some work needs to be done to develop dedicated functionals as the current translated functionals present some risks for double counting. However, overall, we think this method has the potential to revolutionize multiconfigurational quantum chemistry in the same way standard DFT did in quantum chemistry in general.

## Data Availability

All computed values of singlet–triplet gaps are collected in the Supporting Information of this article.

## Supporting Information Available

All computed errors in singlet–triplet gaps (PDF)
