# Supplementary material for: Variational Pair-Density Functional Theory: Dealing with Strong Correlation at the Protein Scale
Source: J Chem Theory Comput. 2024 Jan 13;20(6):2423–32. doi: 10.1021/acs.jctc.3c01240 (PMC10976634; doi:10.1021/acs.jctc.3c01240)
Supplement: Supplementary file 1 — ct3c01240_si_001.pdf [file ct3c01240_si_001.pdf]

Supplementary material:
Variational pair-density functional theory: dealing with strong correlation at the protein scale

Mikael Scott,[1,2] Gabriel Rodrigues,[1] Xin Li,[3] and Mickael Delcey[2]
[1)] *Division of Theoretical Chemistry and Biology, School of Engineering Sciences in Chemistry, Biotechnology and Health, KTH Royal Institute of Technology,SE-100 44 Stockholm, Sweden*
[2)] *Division of Theoretical Chemistry, Department of Chemistry, Lund University, SE-221 00 Lund, Sweden*
[3)] *PDC Center for High Performance Computing, KTH Royal Institute of Technology, SE-100 44 Stockholm, Sweden*

(Dated: 12 January 2024)

			variational ctPDF			perturbative ctPDF			perturbative tPDF		
molecule	active space	CASSCF	ctLDA	ctPBE	ctBLYP	ctLDA	ctPBE	ctBLYP	tLDA	tPBE	tBLYP
O_2	(2,2)	−23.17	−0.88	0.46	0.79	25.03	22.97	21.13	−7.47	−6.66	−6.44
	(6,4)	−3.20	−1.38	−0.42	0.10	4.83	2.91	1.60	−3.76	−3.21	−3.21
O	(2,2)	4.86	−5.25	−2.67	−2.04	−5.38	−2.88	−2.23	−17.15	−15.43	−15.85
OH^+	(2,2)	3.07	−5.95	−3.81	−2.96	−6.64	−3.99	−3.09	−19.52	−17.69	−18.03
NH	(2,2)	5.09	−2.48	−0.54	−0.27	−3.27	−0.70	−0.44	−12.78	−10.96	−11.56
NF	(2,2)	6.26	−6.66	−4.97	−4.76	−5.68	−3.45	−3.20	−15.08	−13.49	−13.93
	(8,6)	2.69	−6.88	−4.86	−4.86	−6.01	−3.87	−3.86	−12.85	−11.19	−11.66
C	(2,2)	5.54	−0.34	2.46	2.32	−0.62	2.08	2.07	−8.69	−6.78	−7.49
Si	(2,2)	5.58	−1.09	1.99	0.75	−1.56	0.90	−0.25	−5.58	−3.76	−4.98
$C_5H_5^+$	(2,2)	−5.62	−4.45	−3.41	−3.44	15.19	14.38	13.97	15.03	14.21	13.80
	(4,5)	7.45	−4.47	−3.39	−1.97	11.58	11.11	10.76	2.49	2.47	2.25
C_4H_2-1,3-2CH_2	(2,2)	−9.24	−10.53	−9.73	−9.77	−1.57	−1.98	−1.86	−1.84	−2.28	−2.17
	(6,6)	−4.21	−10.72	−8.70	−8.99	−3.01	−3.39	−3.45	−3.00	−3.23	−3.42
MAE	(2,2)	7.60	4.18	3.34	3.01	7.22	5.93	5.36	11.46	10.14	10.47
MAE	(π,π^*)	4.94	4.20	3.07	2.58	4.68	3.44	3.04	12.10	10.99	11.24
C_4H_4	(2,2)	10.91	7.24	7.80	7.81	6.79	7.43	7.40	6.12	6.62	6.55
	(4,4)	−6.08	7.11	7.66	7.67	4.70	4.99	5.09	4.23	4.42	4.50
C_4H_3-CHO	(2,2)	6.52	5.72	6.17	6.17	17.20	17.09	17.02	17.04	16.90	16.83
	(6,6)	−6.44	4.25	3.60	3.88	4.68	4.82	4.99	4.09	4.23	4.29
C_4H_3-NH_2	(2,2)	2.09	5.03	5.67	5.54	14.46	14.23	14.00	14.32	14.07	13.84
	(6,5)	−6.04	4.71	5.42	4.78	5.21	5.47	5.51	4.73	4.90	4.92
C_4H_2-NH_2-CHO	(2,2)	−5.30	−8.93	−8.41	−8.41	0.79	0.44	−0.08	0.78	0.43	−0.09
	(8,7)	−1.10	−9.68	−9.27	−9.06	10.25	10.40	10.42	9.88	9.97	9.96
MAE	(2,2)	6.21	6.73	7.01	6.98	9.81	9.79	9.62	9.57	9.50	9.33
MAE	(π,π^*)	4.92	6.44	6.49	6.35	6.21	6.42	6.50	5.73	5.88	5.92

TABLE I. Errors (kcal mol^{-1}) of singlet-triplet energy gaps calculated at the CASSCF , MC-tLDA, MC-tPBE and MC-tBLYP levels with the perturbative and variational approach using the cc-pVTZ basis set. The reference values are described in the main article.
